# Correction: The binding of NCAM to FGFR1 induces a specific cellular response mediated by receptor trafficking

**DOI:** 10.1083/jcb.20090303002222019c

**Published:** 2019-02-26

**Authors:** Chiara Francavilla, Paola Cattaneo, Vladimir Berezin, Elisabeth Bock, Diletta Ami, Ario de Marco, Gerhard Christofori, Ugo Cavallaro

Vol. 187, No. 7, December 28, 2009. 10.1083/jcb.200903030.

Following publication, the authors realized that Fig. 6 A inadvertently contained an incorrect image for the 30-min treatment with FGL in the presence of SU6656 (in the “– acid wash” condition). The image has now been replaced with the correct one (Fig. 6 A below), corresponding to that treatment condition. This correction does not change the conclusions of the experiment, namely, that little or no localization of HA-FGFR1 on the cell surface was detected upon treatment with either FGF2 or FGL upon Src inhibition. The authors regret any confusion this may have caused.

**Figure 6.**
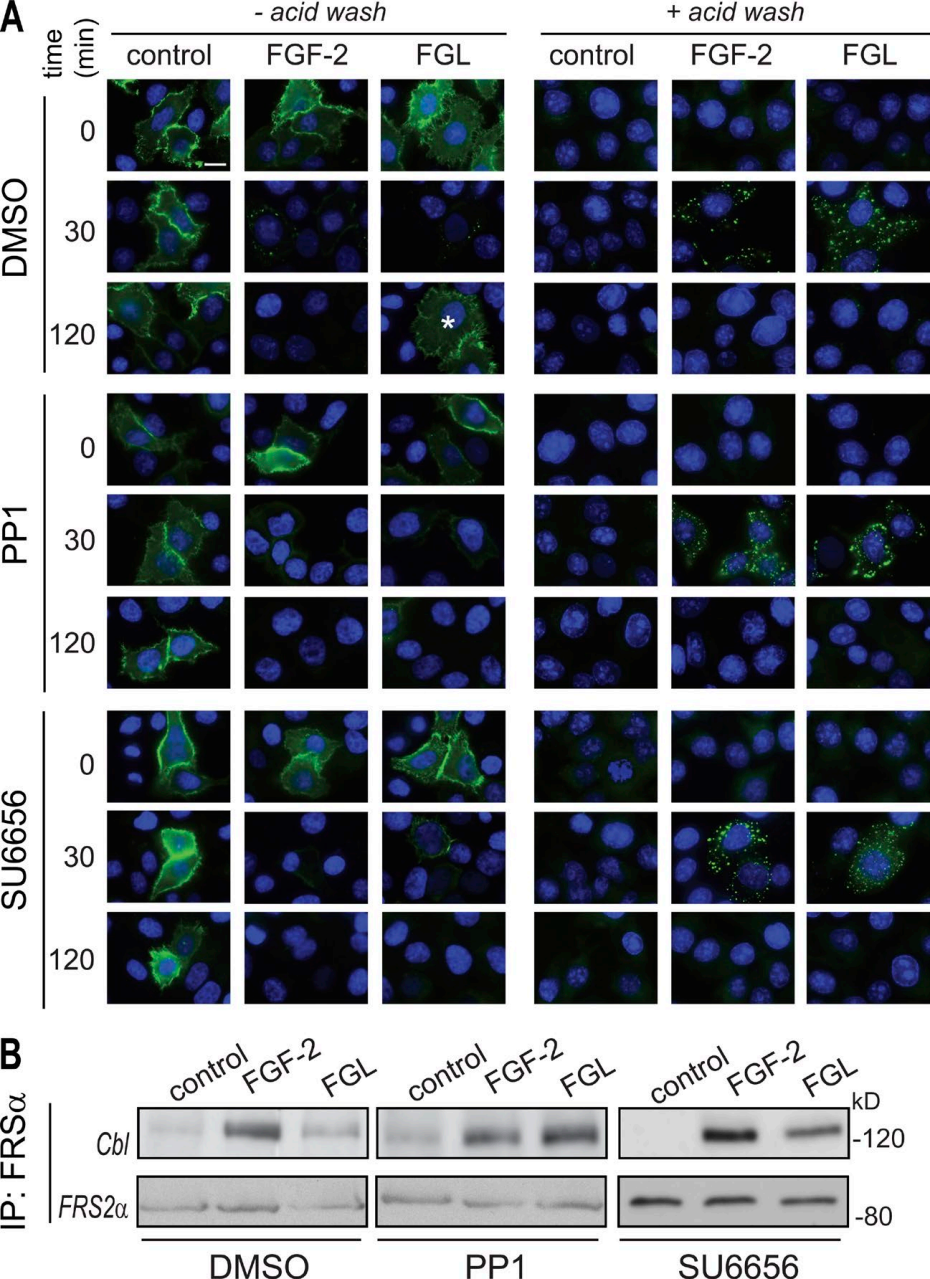
**Src inhibition blocks the recycling of FGFR1 and promotes the association of Cbl with FRS-2α in NCAM-stimulated cells.** (A) HeLa cells stimulated with FGF-2 or FGL in the presence of DMSO (top), PP1 (middle), or SU6656 (bottom) were processed as for Fig. 3 A. Asterisk marks a cell where HA-FGFR1 recycled back to the cell surface. Bar, 10 µm. (B) HeLa cells were treated with DMSO (left), PP1 (middle), or SU6656 (right) before stimulation with either FGF-2 or FGL for 10 min. Cell extracts were immunoprecipitated (IP) with anti–FRS-2α antibody and immunoblotted for Cbl (top) followed by immunoblotting for FRS-2α (bottom).

